# Purification and Characterization of Iso-Ribonucleases from a Novel Thermophilic Fungus

**DOI:** 10.3390/ijms15010944

**Published:** 2014-01-10

**Authors:** Kyle S. Landry, Robert E. Levin

**Affiliations:** Department of Food Science, Massachusetts Agricultural Experiment Station, University of Massachusetts, Amherst, MA 01003, USA; E-Mail: kslandry@foodsci.umass.edu

**Keywords:** affinity purification, RNase purification, chromatography, thermophilic fungi, RNase characterization

## Abstract

A thermophilic fungus previously isolated from composted horse manure was found to produce extracellular iso-RNases that were purified 127.6-fold using a combination of size exclusion chromatography and a novel affinity membrane purification system. The extent of purification was determined electrophoretically using 4%–15% gradient polyacrylamide gels. RNase activity was dependent on the presence of a metal co-factor with significantly more activity with Zn^2+^ or Mn^2+^ than Mg^2+^. The RNases exhibited maximum activity at both pH 3.0 and pH 7.0 with no activity at pH 2.0 or 10.0. The optimal temperature for the iso-RNase was 70 °C. The molecular weight of the iso-RNase was determined to be 69 kDa using a Sephadex G-75 column.

## 1. Introduction

Ribonucleases (RNase) are found in all organisms including animals, plants and microorganisms and were the first nucleases to be described [1]. Ribonuclease A was first purified and crystallized from beef pancreas by Kunitz [2]. Ribonucleases can hydrolyze single stranded or double stranded RNA as well as RNA present as a DNA-RNA hybrid [3]. Bovine pancreatic ribonuclease is the most characterized of all nucleases and was the first enzyme to be subjected to sequencing, protein crystallographic, and protein folding studies [4]. Currently there are hundreds of known RNases that have been fully or partially characterized, with many more to be discovered.

Recently a thermophilic fungus was isolated and found to produce an extracellular DNase [5]. It was later found that the isolate also produces an extracellular RNase. This manuscript describes the purification and characterization of the extracellular RNase produced by the isolate.

## 2. Results and Discussion

### 2.1. Concentration of Crude RNase

Following filtration and dialysis of the culture, the enzyme activity of the crude sample was analyzed for RNase activity using the acid soluble assay at 55 °C. The crude sample (250 mL) was then concentrated at room temperature to 39 mL using an ultra-filtration apparatus with a membrane cut off of 10 kDa, resulting in a concentration factor of 6.41.

**Figure 1.**
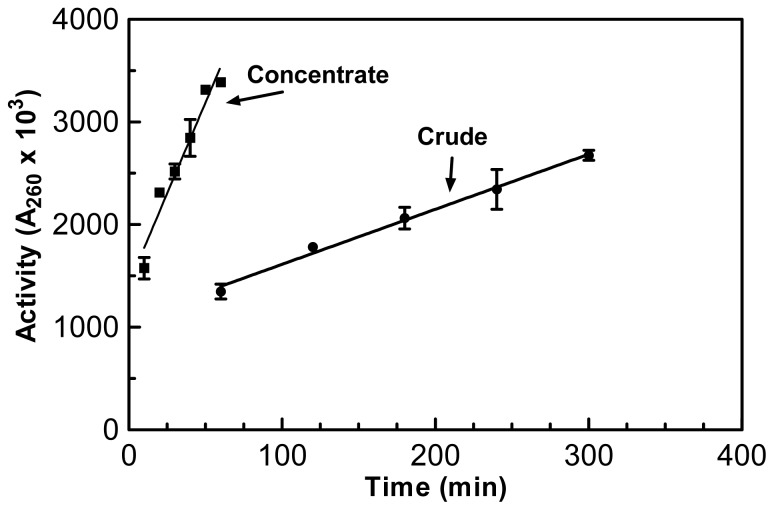
Comparison of the rates or slopes of the crude and concentrated samples in regards to enzyme activity. The assay used for these experiments was the acid soluble assay which was performed in triplicate at 55 °C.

Enzyme activities from both the crude and concentrated enzyme preparations were plotted and their slopes analyzed (Figure 1). If no enzyme activity was lost during the concentration then the rate or slope of the concentrated sample when divided by the concentration factor should equal that of the crude preparation.

The rate of activity of the concentrated enzyme, when divided by the concentration factor was 98% of the original crude activity slope, indicating that only 2% of the total enzyme activity was lost during the concentration process (Table 1). Overall there was a 9.07-fold increase in specific activity when compared to the original crude sample based on the Lowry assay. In regards to overall protein, the crude sample had 320 mg of protein and after membrane concentration; the overall protein content was reduced by 89% to 35 mg according to the Lowry assay (Table 1).

### 2.2. Sephadex G-50 Purification

**Figure 2.**
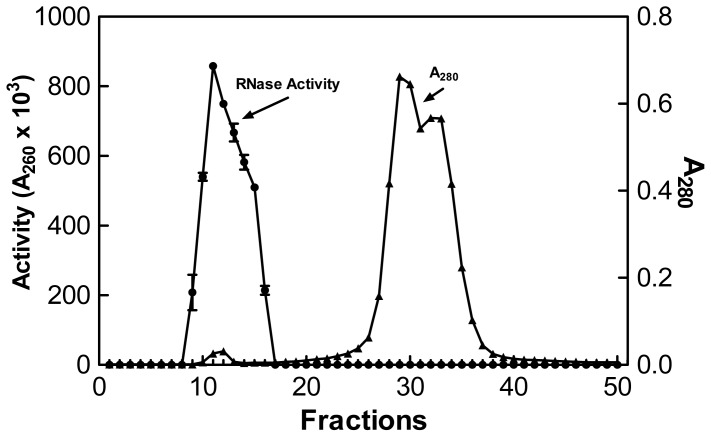
Typical plots of protein and enzyme activity when 1 mL of concentrated sample was passed through a Sephadex G-50 column at 2–5 °C. The activity was measured using the acid soluble assay at 55 °C and protein was estimated by determining the absorbance at 280 nm.

The next stage of purification involved passing the concentrated sample through a Sephadex G-50 column in refrigerated chamber (2–5 °C). The void volume for the column was determined to be 52.8 mL using Blue Dextran 2000 (GE Healthcare Cat# 45-000-048; Hertfordshire, UK). The first fraction demonstrating a measureable absorbance reading at 280 nm was fraction 9 with additional readings extending to fraction 50. Fractions 9 through 16 had RNase activity (Figure 2). All tubes with RNase activity were pooled. The remaining fractions demonstrated no activity. Purification via Sephadex G-50 resulted in a 91.5-fold increase in specific activity. A typical plot of protein and activity for RNase can be seen in Figure 2.

### 2.3. Membrane Purification

Following Sephadex G-50 chromatography, only two protein bands remained, one of which was known to be DNase [5]. The membrane purification system developed by Landry and Levin [6] was then utilized to separate the RNase form the DNase.

**Figure 3.**
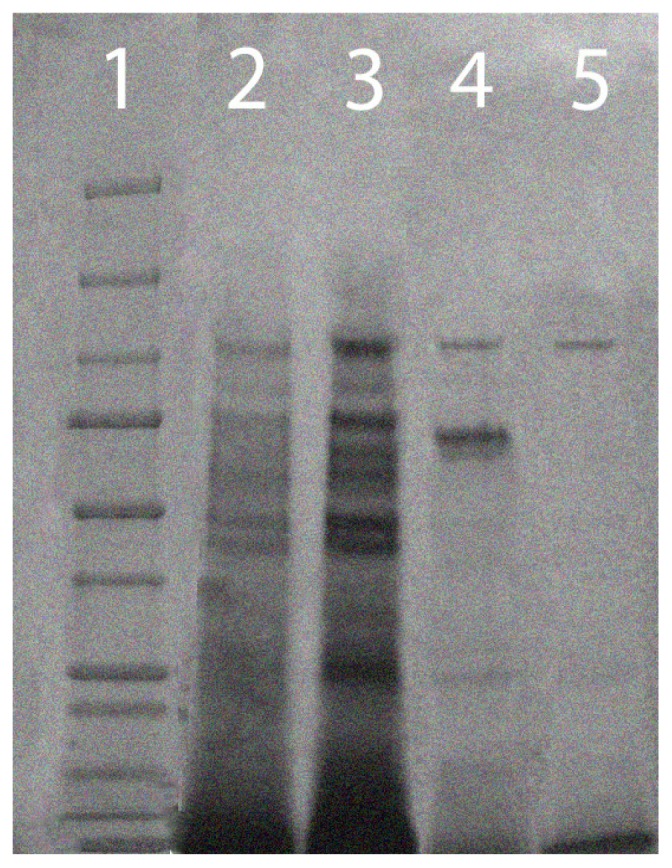
Enzyme purification was determined using 4%–15% gradient polyacrylamide gel electrophoresis. The lanes are as follows: (1) 250–10 kDa protein ladder; (2) crude sample; (3) concentrated sample; (4) Sephadex G-50 sample; and (5) purified RNase.

Pooled fractions from four Sephadex G-50 refrigerated columns (211 mL) were concentrated to 45 mL using the pressurized ultrafiltration cell as described earlier. From this preparation, 10 mL were purified using 10 DNA coated membranes. This was done for the remaining 35 mL as well. Following membrane purification, the purified sample was assayed for activity and protein concentration. Membrane purification resulted in the retention of 76% of the initial activity with a 121-fold increase in specific activity (Table 1). The overall protein content of the membrane purified RNase was 168 times less than that of the original crude sample, with the final concentration of protein being just under 2 mg (Table 1). Enzyme purity was assessed using 4%–15% gradient polyacrylamide gels. The purification of the RNase can be seen in Figure 3.

### 2.4. Effect of Temperature on the Partially Purified RNase

**Figure 4.**
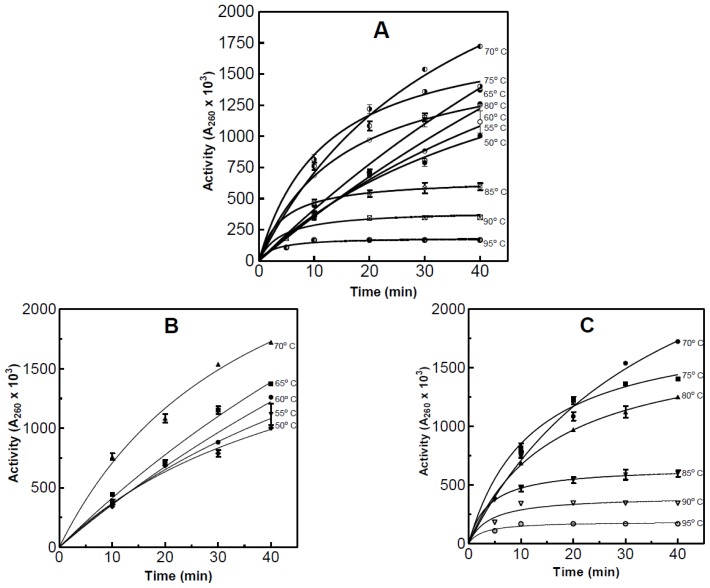
The effect of temperature on the activity of the partially purified RNase. The plots are as follows: (**A**) plot of all of the temperatures assayed for activity; (**B**) plot of the temperatures 50–70 °C; and (**C**) plot of the temperatures 70–95 °C. Activity was measured using the acid soluble assay which was performed in triplicate.

The effect of temperature on enzyme activity of the partially purified RNase was assessed two ways. The first method involved using the acid soluble assay after various reaction temperatures. Using this method the optimum and maximum temperatures for enzyme activity was determined to be 70 and 95 °C respectively, with no detectable activity 95 °C (Figure 4). The activity at the optimum temperature was 73% greater than at 50 °C.

**Figure 5.**
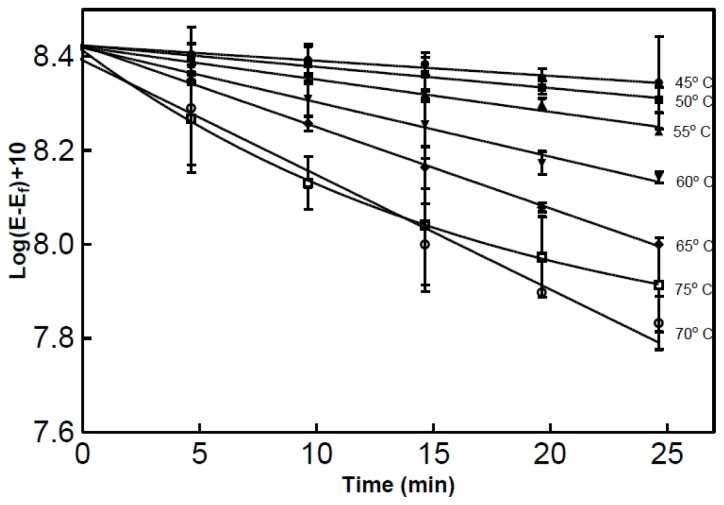
Effect of temperature on partially purified RNase activity. Measurements were in triplicate using a modified version of the real-time assay developed by Kunitz [7].

The second method used a modified version of the real-time spectrophotometric assay developed by Kunitz [7]. 1.0 mL of partially purified RNase was added to 2.0 mL of substrate and the decrease in absorbance at 300 nm was recorded as seen in Figure 5. Prior to analysis both the sample and buffer were equilibrated to the desirable temperature. Using this method the optimum temperature was again found to be 70 °C however the maximum temperature was not assessed using this method.

**Figure 6.**
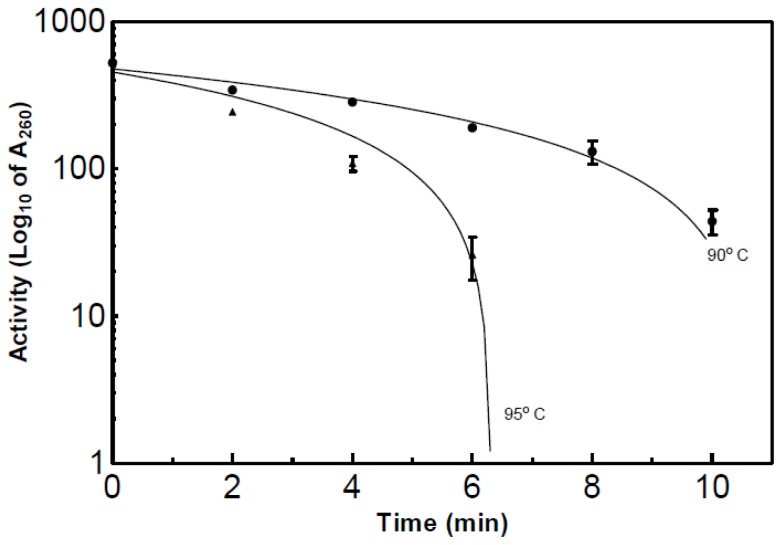
Plots of the denaturation of partially purified RNase at 90 and 95 °C. The assay used for this experiment was a modified version of the acid soluble assay which is explained in detail in the Materials and Methods Section.

The rate of thermal denaturation was assed at 90 and 95 °C. These two temperatures were selected from the results seen in Figure 4C. The denaturation plot can be seen in Figure 6. An enzyme sample exposed to 90 °C for 10 min resulted in a 92% decrease in activity when compared to the initial activity. After 7 min at 95 °C, 100% of the activity was lost, while enzyme exposed for 6 min to 95 °C lost 95% of the activity.

### 2.5. Effect of pH on the Partially Purified RNase

The activity of the partially purified RNase was determined at various pH values between pH 2.0 and 10.0. A modified version of the acid soluble assay was used which involved adding 0.75 mL of partially purified RNase to 0.75 mL of substrate (yeast RNA; 1 mg/mL (Sigma Cat# R6625; Milwaukee, WI, USA), 10 μmol MgSO_4_ in a 0.1 M imidazole, malic acid, and glycine buffer, at various pH) and allowed to incubate at 55 °C for 10 min. The rest of the procedure was carried out as described in the Materials and Methods section.

**Figure 7.**
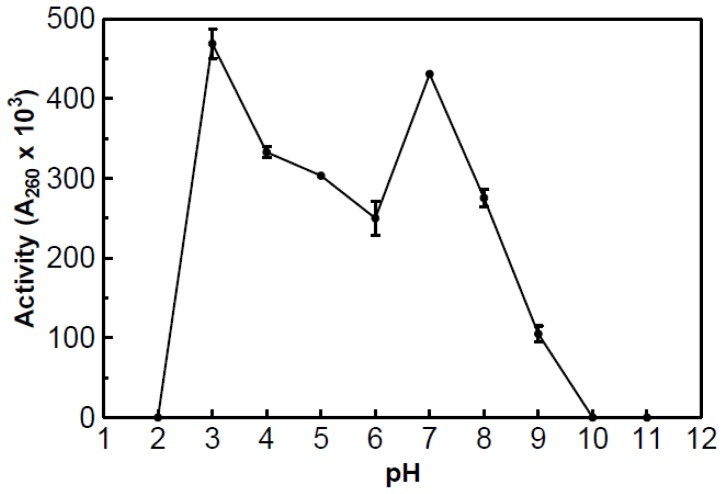
Plot of the partially purified RNase pH profile. The activity was measured using a modified version of the acid soluble assay at 55 °C with varying pH.

The partially purified RNase demonstrated activity throughout the pH range of 3.0–9.0 and exhibited optimum activity at both pH 3.0 and 7.0 (Figure 7). No activity was detected at pH 2.0 or 10.0. The presence of two optimal pH peaks may indicate the presence of iso- RNases, which is why it is to be considered partially purified.

### 2.6. Effect of Dialysis and EDTA on RNase Activity

**Figure 8.**
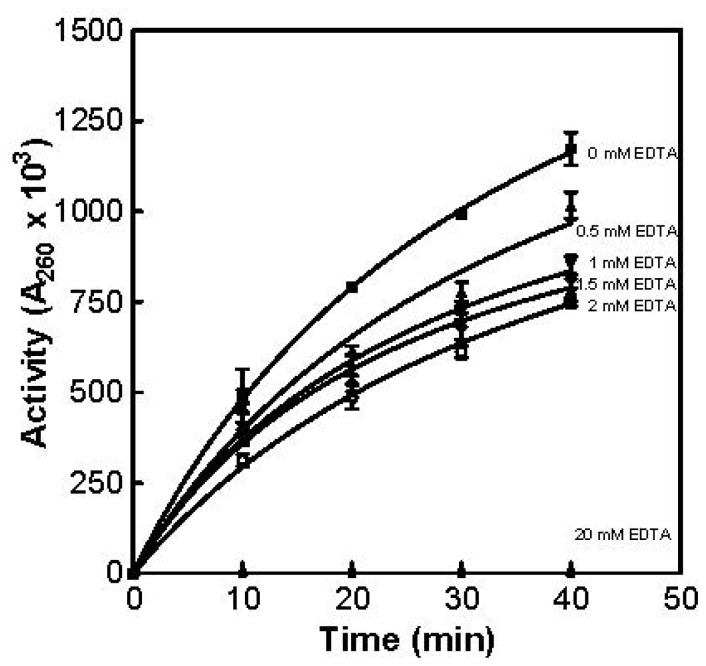
The effect of EDTA on partially purified RNase activity. The results were obtained using the acid soluble assay at 55 °C with the designated amount of EDTA added to the substrate.

Partially purified RNase was dialyzed by filtering the sample with 500 mL of deionized water via ultrafiltration as described in the Materials and Methods section. Care was taken to ensure that the final sample volume was the same as the initial sample volume prior to dialysis filtration. It was found that there was no significant difference in activity between dialyzed and non-dialyzed sample. The effect of EDTA on enzyme activity was also examined. Partially purified RNase (0.75 mL) was added to 0.75 mL of substrate (yeast RNA; 1 mg/mL (Sigma Cat# R6625; Milwaukee, WI, USA) in 0.1 M imidazole buffer (pH 7.0) containing 2 mM MgSO_4_, plus 0 mM, 0.5 mM, 1.0 mM, 1.5 mM, or 2.0 mM Na_2_·EDTA) and incubated in a water bath set to 55 °C. The reaction was carried out as previously described. With 2.0 mM of Na_2_·EDTA present in the substrate, a 35% decrease in enzyme activity was observed. All activity was inhibited when 20 mM of Na_2_·EDTA was added to the substrate (Figure 8).

### 2.7. Effect of Metal Ions on Enzymatic Activity

**Figure 9.**
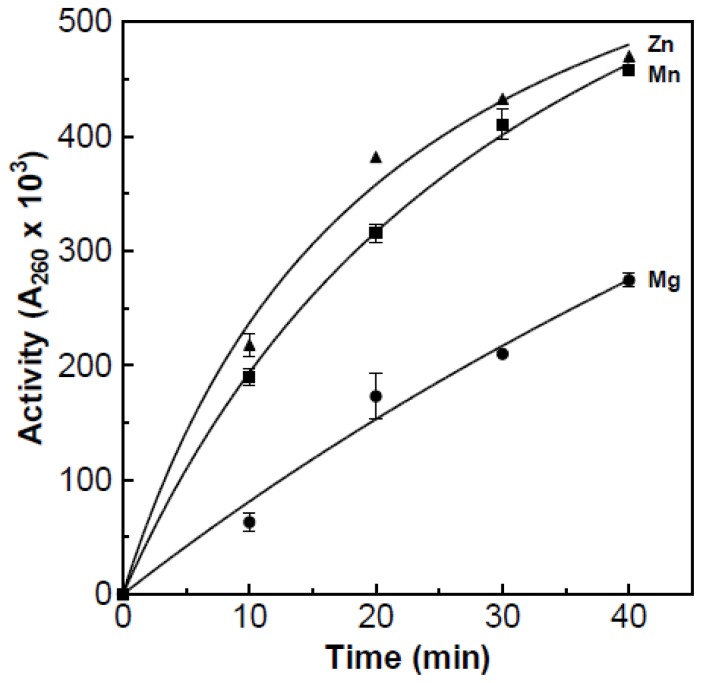
The effect of various metal ions on partially purified RNase activity. The results were obtained using the acid soluble assay at 55 °C with 50 mM/mL of the designated metal ions added to the substrate. Prior to the experiment 20 mM/mL of EDTA was added to the partially purified RNase.

Partially purified RNase stripped of its metal co-factors by the addition of 20 mM Na_2_·EDTA. This was used to determine the effect of different metal ions on enzyme activity. The ions tested were zinc (as zinc acetate, Fisher Scientific, Cat# Z20-500; Pittsburgh, PA, USA) magnesium (as magnesium sulfate, Fisher Scientific, Cat# M65-500; Pittsburgh, PA, USA), and manganese (as manganese chloride, Fisher Scientific, Cat# M87-100; Pittsburgh, PA, USA); the desired metal ion (50 mM) was added to the substrate to ensure that the metal ions would not be sequestered by unbound EDTA. The acid soluble assay at 55 °C was used to measure the effect of metal ions on enzyme activity (Figure 9). The metal ions zinc and manganese both resulted in the highest amount of activity. The magnesium ion resulted in about 50% of the activity achieved with the zinc and manganese ions.

### 2.8. Estimated Molecular Weight of the Partially Purified RNase

**Figure 10.**
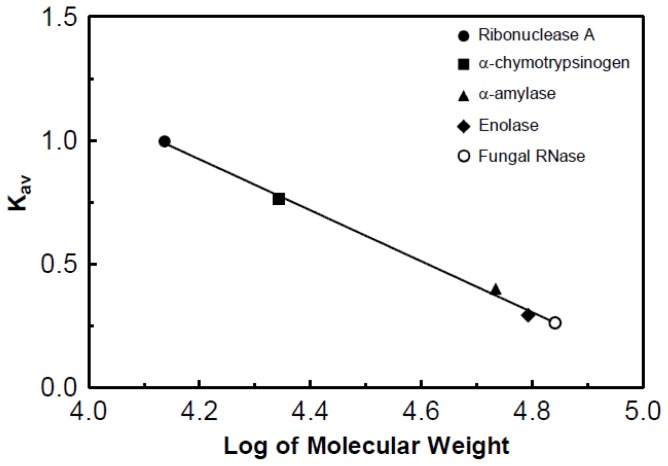
Molecular weight determination of the partially purified RNase. Molecular weights of protein standards were: ribonuclease A, 13,700; α-chymotypsinogen, 25,000; α-amylase, 51,000; and enolase, 82,000. The log molecular weight of the RNase was determined to be 4.841, which is roughly equal to a molecular weight of 69 kDa.

The molecular weight of the purified DNase was estimated using a column of Sephadex G-75. The calculated *K*_av_ values were plotted against the log molecular weight of the standard proteins. Chromatography of the RNase yielded a log molecular weight of 4.84 and therefore the partially purified RNase has an estimated molecular weight of 69,000 (Figure 10).

### 2.9. *K*_m_ and *V*_max_ of the Partially Purified RNase

**Figure 11.**
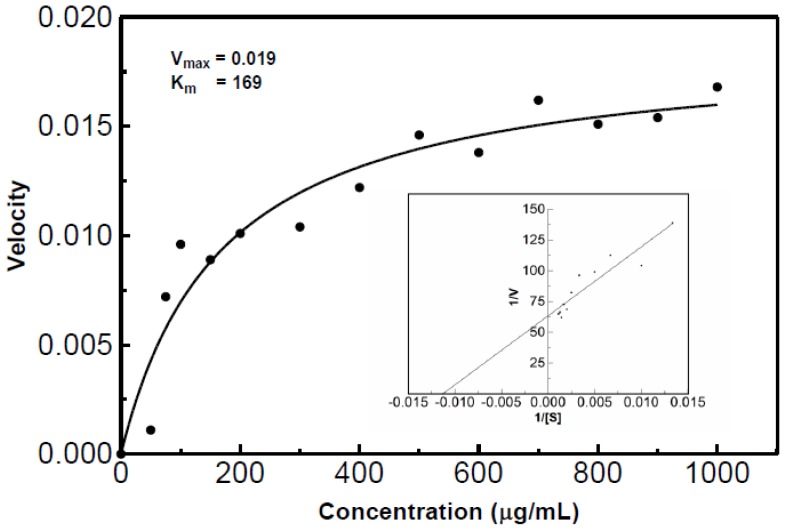
Michaelis-Menten and Lineweaver-Burk plots of RNase activity. The *K*_m_ value was estimated to be 169 with a *V*_max_ of 0.019. The acid soluble assay at 55 °C with varying RNA substrate concentrations was used to determine the velocities. The data was then converted to generate the Lineweaver-Burk plot.

The *K*_m_ and *V*_max_ for the partially purified RNase was estimated by plotting the velocities against the substrate concentration (Figure 11). The velocities for each concentration were generated using the acid soluble assay at 55 °C with varying concentrations of RNA. The RNase was estimated to have a *K*_m_ value of 169 and a *V*_max_ value of 0.019.

## 3. Materials and Methods

### 3.1. Isolation of the Thermophilic Fungus

The strain (TM-417) used in this manuscript was originally isolated by this laboratory [5].

### 3.2. Initial RNase Screening

TM-417 was inoculated on yeast protein soluble starch media plates (YpSs; 0.4% yeast extract, 0.1% K_2_HPO_4_, 0.05% MgSO_4_, 1.5% soluble starch, pH 7.3) that was modified according to Korn *et al*. [8] utilizing the dye thionine to detect the presence of an extracellular ribonuclease (RYpSs). RNase activity was indicated by the presence of a pink halo around the isolate.

### 3.3. Effect of Temperature on RNase Production

A correlation between temperature and RNase production was established by inoculating RYpSs agar plates with a 10 mm plug taken from a stock culture plate. The plates were incubated at various temperatures (2, 20, 32, 37, 45, 55, and 60 °C) for five days. The zones of clearing in mm were measured in four different directions from the edge of visible growth to the furthest spot of clearing.

### 3.4. Preparation of Sample for RNase Purification

Isolated cultures were transferred to Fernbach flasks containing 500 mL of YpSs broth containing 0.2% yeast RNA (Sigma Cat# R6625; Milwaukee, WI, USA). Each flask was placed on a rotary shaker set to 115 rpm and incubated at 55 °C. After three days the flasks were allowed to incubate statically for an additional four days. Cell mass was removed from the culture by vacuum filtration through coarse filter paper (Fisher Scientific Filter Paper P8 Cat# 09-790-12D; Pittsburgh, PA, USA). The filtrate was filtered under vacuum through medium (Fisher Scientific Filter Paper P5 Cat# S47574D; Pittsburgh, PA, USA), and then through fine filter paper (Fisher Scientific Filter Paper P2 Cat# S47574D; Pittsburgh, PA, USA). The filtered sample represented the crude enzyme. To inhibit any bacterial and/or fungal growth, 0.02% sodium azide was added to the crude enzyme sample. All crude enzyme samples were stored at ambient temperature.

### 3.5. Sample Purity

Electrophoresis was carried out in a vertical Bio-Rad Mini-Protean^®^ system using a Tris/Glycine/SDS buffer (Bio-Rad Cat# 161-0772; Hercules, CA, USA). The sample was diluted 1:1 with a Lamemmli buffer solution containing 950 μL Lamemmli buffer and 50 μL β-mercaptoethanol Lamemmli buffer (Bio-Rad Cat# 161-0737; Hercules, CA, USA)/β-mercaptoethanol (Fisher Scientific Cat# O3446I-100; Pittsburgh, PA, USA). The mixture was heated for 5 min at 99 °C. Proteins were separated using 4%–15% gradient Mini-Protean^®^ TGX™ 30 μL well precast gels (Bio-Rad Cat# 456-1083; Hercules, CA, USA). Gels were loaded with 30 μL of sample and run at 155 V for 45 min. Gels were placed on a shaker set to low speed (25 RPM) and stained using 50 mL of Acqua Stain (Bulldog Bio Co. Cat# AS001000; Portsmouth, NH, USA) for 60 min. The bands were photographed with a PowerShot G10 Digital Canon Camera (Tokyo, Japan) equipped with an orange filter. A 250–10 kDa protein ladder (Dual Color Precision Plus Protein Standards. Bio-Rad Cat# 161-0374; Hercules, CA, USA) used was as a standard.

### 3.6. Determination of Protein Content

Protein was determined by the Lowry method [9] or by measuring absorbance at 280 nm. A blank was generated by substituting the sample with deionized water.

### 3.7. Acid Soluble Assay for Enzyme Activity

RNase activity was determined by measuring acid soluble nucleic acids. The method used in this study was a modified version of the method presented by Eaves and Jeffrie [10]. Enzyme sample (0.75 mL) was added to 0.75 mL of substrate (yeast RNA, Sigma Cat# R6625; Milwaukee, WI, USA, 10 μmol MgSO_4_ in 0.1 M imidazole buffer, pH 7.0) and incubated in a controlled water bath at 25 °C for 10 min. The reaction was stopped by adding 0.5 mL of uranylacetate-perchloric acid reagent (0.25% uranylacetate in 10% perchloric acid). Reaction tubes were cooled in an ice bath for 15 min. The mixture was diluted with 2.0 mL of deionized water and the precipitate removed by centrifugation at 13,400 rpm for 5 min at ambient temperature. The absorption at 260 nm was then measured against a reagent blank prepared by adding the uranylacetate-perchloric acid reagent to the substrate prior to the addition of the enzyme. One unit of enzyme activity is defined as an increase in absorbance of 0.1 units in a cuvette of 1 cm light path at 260 nm.

### 3.8. Ultrafiltration Membrane Concentration

Crude enzyme sample (250 mL) was dialyzed against deionized water (containing 0.02% sodium azide) for 12 h using dialysis tubing (Fisher Scientific Cat# 21-152-5; Pittsburgh, PA, USA) with a flat width of 40 mm and a molecular weight cut-off of 6–8 kDa.

Prior to sample ultrafiltration, 200 mL of distilled water followed by 100 mL of 1% (*w*/*v*) bovine serum albumin (Fisher Biotech, Fraction V, Cat# BP1605100; Pittsburgh, PA, USA) solution was passed through the pressure cell/membrane apparatus. This was to ensure that no protein from the sample would bind to the membrane. The dialyzed crude enzyme sample was then concentrated using a 500 mL pressure cell (Amicon^©^ Cat# 5124; Billerica, MA, USA) and a 10 kDa cellulose membrane (Millipore^©^ Utrafiltration YM10 Dia. 76 mm, Cat# 13642; Billerica, MA, USA) yielding the concentrated enzyme sample.

### 3.9. Sephadex G-50 Column Chromatography

Sephadex G-50 (MP Biomedicals Cat# ICN19558010; Solon, OH, USA) was hydrated in deionized water for 3 h at 100 °C prior to loading the column. A 45 cm × 2.5 cm glass column was used. A 34 cm long column of Sephadex G-50 was equilibrated for 24 h with 0.5 M imidazole buffer (pH 7.0) containing 0.02% sodium azide. A portion (1.0 mL) of concentrated sample was loaded onto the bottom of the gel bed and eluted ascendingly with the 0.5 M imidazole buffer at a rate of 2 mL/min in a refrigerated chromatography cabinet (2–5 °C). Fractions (6.6 mL) were collected using a Gilson FC 203B fraction collector (Middleton, WI, USA). All fractions which demonstrated RNase activity were pooled together. Activity was measured using the acid soluble assay at 55 °C with an incubation time of 20 min.

### 3.10. Membrane Preparation for Affinity Purification

A 47 mm, 0.2 μm FP-Vericel membrane (Pall Life Sciences Cat# 66477; Radnor, PA, USA) was inserted into a Millipore^©^ membrane filtration unit and rinsed under vacuum with 200 mL of deionized water. One gram of salmon sperm DNA (USB Cat# 14405 100 GM; Santa Clara, CA, USA) was dissolved in 50 mL of 2× SSC buffer (1.75% sodium chloride, 0.88% sodium citrate, adjusted to pH 7.0) and boiled for one minute. The boiled solution was placed in ice and cooled to 10 °C. The rinsed membrane was added to the solution and placed in an iced bucket. The iced bucket was placed on a shaker at 75 RPM. After 60 min the membrane was removed, rinsed with deionized water and dried using a stream of hot air.

### 3.11. Affinity Membrane DNase Separation

10 mL of sample from four concentrated Sephadex G-50 column runs (45 mL total volume) was chilled to 5 °C in a 200 mL beaker and place in an iced bucket on a shaker set to 50 rpm in a 2 °C refrigerator. DNA coated membranes were prepared according to Landry and Levin [6] and added to the chilled sample and removed after 3 min and then removed. The remaining 35 mL of concentrated Sephadex G-50 sample was purified in the same manner. All membranes were discarded after one use.

### 3.12. Spectrophotometric Assay of RNase Activity

The method used for this study was a modified version of the one used by Kunitz [7]. Approximately 2.25 mL of substrate (yeast RNA; 1 mg/mL (Sigma Cat# R6625; Milwaukee, WI, USA), 10 μmol MgSO_4_ in 0.1 M imidazole, pH 7.0) was added to a cuvette (1 cm path length) and placed into a temperature controlled cuvette holder and equilibrated to the desired temperature. Partially purified enzyme (0.75 mL) was added and mixed with a cuvette mixing device, for a total volume of 3 mL. The decrease in absorbance at 300 nm was recorded. The blank consisted of 2.25 mL of substrate solution and 0.75 mL of deionized water. The recorded absorbance was then converted to log(*E* − *E_f_*) +10 where *E* = *A*_300_ and *E_f_* = *A*_300_ after 30 min of digestion.

### 3.13. Enzyme Destruction Assay

Two water baths were used for this assay, one set to 55 °C and another set to 90 and 95 °C. The enzyme sample was placed in the water bath set to the desired temperature and allowed to equilibrate. Once the enzyme preparation reached the desired temperature, the enzyme (0.75 mL) was removed and added to 0.75 mL of substrate (yeast RNA; 1 mg/mL (Sigma Cat# R6625; Milwaukee, WI, USA), 10 μmol MgSO_4_ in 0.1 M imidazole, pH 7.0) equilibrated at 55 °C. The incubation time was 10 min. The enzyme sample was tested at various time intervals during the incubation at the desired temperature.

### 3.14. Molecular Weight Determination via Sephadex G-75 Chromatography

The molecular weight was determined using a column of Sephadex G-75 (Pfaltz & Bauer Inc. Cat# 50-145-149; Waterbury, CT, USA) with a gel bed of 2.5 × 30 cm and a pH 7, 0.5 M imidazole buffer containing 0.02% sodium azide. The flow rate for the column was 0.8 mL/min (48 mL/h). The *K*_av_ values of enzymes with known molecular weights were used to determine the molecular weight from a standard curve. Highly purified ribonuclease A (13,700 Da; MP Biomedicals Cat# ICN19398050; Solon, OH, USA), α-chymotrypsinogen (25,000 Da; Crescent Chemical Co. Cat# 50-247-444; Islandia, NY, USA), α-amylase (51,000 Da; Fisher Scientific Cat# S25135; Pittsburgh, PA, USA), and enolase (82,000 Da; MP Biomedicals Cat# ICN1934989; Solon, OH, USA) as well as Blue Dextran 2000 (GE Healthcare Cat# 45-000-048; Hertfordshire, UK) were used to generate the standard curve.

## 4. Conclusions

In sum, a thermophilic RNase was partially purified and characterized in terms of its optimal conditions, molecular weight, and certain kinetic properties. One of the most interesting findings regarding this nuclease is its activity profile at near boiling conditions. This observation is not surprising since the maximum temperatures from thermophilic organisms should theoretically be higher than other mesophiilic enzymes.

It should be noted that some RNases from the fungi kingdom have been shown to exhibit angiogenic, antitumor, and immunosuppressive activities [11–13]. These findings have increased the interest in fungal RNase purification and characterization. Yet, there has been very little, if any research done regarding nucleases from thermophilic fungi. RNases with potential medical benefits are largely from edible mushrooms belonging to the phyla basidiomycota [13]. The presented RNase was isolated from a thermophilic *Chaetomium* sp. [5]. Filamentous ascomycetes may produce extracellular nucleases that may be as promising, if not better than currently studied fungi.

## Figures and Tables

**Table 1.** Overall purification of the thermophilic RNase a.

Fraction	Vol (mL)	Protein Content (mg)	Total activity (U)	Sp act (U/mg)	Recovery (%)	Increase in sp act
Crude	250.0	320.0	10,476	33	100	1
Concentrated	39.0	35.0	10,257	293	98	8.9
Sephadex G-50	46.2	2.8	8456	3020	81	91.5
Membrane	45	1.9	8006	4214	76	127.6

a One unit of enzyme activity is defined as an increase in absorbance of 0.1 units in a cuvette of 1 cm path length at 260 nm.
